# Development of a novel methodology for ascertaining scientific opinion and extent of agreement

**DOI:** 10.1371/journal.pone.0313541

**Published:** 2024-12-06

**Authors:** Peter Vickers, Ludovica Adamo, Mark Alfano, Cory Clark, Eleonora Cresto, He Cui, Haixin Dang, Finnur Dellsén, Nathalie Dupin, Laura Gradowski, Simon Graf, Aline Guevara, Mark Hallap, Jesse Hamilton, Mariann Hardey, Paula Helm, Asheley Landrum, Neil Levy, Edouard Machery, Sarah Mills, Seán Muller, Joanne Sheppard, Shinod N. K., Matthew Slater, Jacob Stegenga, Henning Strandin, Michael T. Stuart, David Sweet, Ufuk Tasdan, Henry Taylor, Owen Towler, Dana Tulodziecki, Heidi Tworek, Rebecca Wallbank, Harald Wiltsche, Samantha Mitchell Finnigan

**Affiliations:** 1 Department of Philosophy, University of Durham, Durham, United Kingdom; 2 School of Philosophy, Religion and History of Science, University of Leeds, Leeds, United Kingdom; 3 Department of Philosophy, Macquarie University, Sydney, Australia; 4 The Wharton School, University of Pennsylvania, Philadelphia, Pennsylvania, United States of America; 5 School of Arts and Sciences, University of Pennsylvania, Philadelphia, Pennsylvania, United States of America; 6 Institute for Philosophical Research - SADAF, National Council for Scientific and Technical Research (CONICET), Buenos Aires, Argentina; 7 Department of Philosophy, University of Nebraska Omaha, Omaha, Nebraska, United States of America; 8 Faculty of Philosophy, History, and Archeology, University of Iceland, Reykjavik, Iceland; 9 Department of Philosophy, Law, and International Studies, Inland Norway University of Applied Sciences, Lillehammer, Norway; 10 Department of Philosophy, Classics, History of Art and Ideas, University of Oslo, Oslo, Norway; 11 School of Social and Political Science, University of Edinburgh, Edinburgh, United Kingdom; 12 Department of History and Philosophy of Science, University of Pittsburgh, Pittsburgh, Pennsylvania, United States of America; 13 Science Communication Unit, Institute of Nuclear Sciences, National Autonomous University of Mexico (UNAM), Mexico City, Mexico; 14 Department of Philosophy, University of Toronto, Toronto, Canada; 15 Department of Philosophy, University of Pennsylvania, Philadelphia, Pennsylvania, United States of America; 16 Durham University Business School, University of Durham, Durham, United Kingdom; 17 Department of Media and Culture, University of Amsterdam (UvA), Amsterdam, Netherlands; 18 Walter Cronkite School of Journalism and Mass Communication, Arizona State University, Phoenix, Arizona, United States of America; 19 Uehiro Oxford Institute, University of Oxford, Oxford, United Kingdom; 20 Johannesburg Institute for Advanced Study, University of Johannesburg, Johannesburg, South Africa; 21 Janelia Research Campus, Howard Hughes Medical Institute, Ashburn, Virginia, United States of America; 22 Department of Philosophy, University of Hyderabad, Hyderabad, India; 23 Department of Philosophy, Bucknell University, Lewisburg, Pennsylvania, United States of America; 24 Leverhulme Centre for the Future of Intelligence, University of Cambridge, Cambridge, United Kingdom; 25 School of Humanities, Nanyang Technological University (NTU), Singapore, Singapore; 26 Department of Philosophy, Stockholm University, Stockholm, Sweden; 27 Department of Philosophy, University of York, York, United Kingdom; 28 Department of Emergency Medicine, University of British Columbia, Vancouver, Canada; 29 Department of Philosophy, University of Birmingham, Birmingham, United Kingdom; 30 Department of Philosophy, Purdue University, West Lafayette, Indiana, United States of America; 31 Department of History and School of Public Policy and Global Affairs, University of British Columbia, Vancouver, Canada; 32 Department of Philosophy, Uppsala University, Uppsala, Sweden; 33 Division of Philosophy and Applied Ethics, Linköping University, Linköping, Sweden; 34 Advanced Research Computing, University of Durham, Durham, United Kingdom; Lahore Medical and Dental College, PAKISTAN

## Abstract

We take up the challenge of developing an international network with capacity to survey the world’s scientists on an ongoing basis, providing rich datasets regarding the opinions of scientists and scientific sub-communities, both at a time and also over time. The novel methodology employed sees local coordinators, at each institution in the network, sending survey invitation emails internally to scientists at their home institution. The emails link to a ‘10 second survey’, where the participant is presented with a single statement to consider, and a standard five-point Likert scale. In June 2023, a group of 30 philosophers and social scientists invited 20,085 scientists across 30 institutions in 12 countries to participate, gathering 6,807 responses to the statement *Science has put it beyond reasonable doubt that COVID-19 is caused by a virus*. The study demonstrates that it is possible to establish a global network to quickly ascertain scientific opinion on a large international scale, with high response rate, low opt-out rate, and in a way that allows for significant (perhaps indefinite) repeatability. Measuring scientific opinion in this new way would be a valuable complement to currently available approaches, potentially informing policy decisions and public understanding across diverse fields.

## Introduction

Attempts to measure scientific opinion are not uncommon [1–5]. In some cases, we know in advance that a scientific consensus (probably) exists, but we also know that many people are unaware of the consensus, or even believe that relevant experts are significantly divided when they are not [6–8]. In some cases there is value in assessing the strength of agreement, or consensus, on a specific topic. In other cases, there may be value in revealing variations in scientific opinion across geographical regions, across fields of expertise, or across time. Survey methods are a valuable tool, but as things stand very limited means for scientific agreement assessments are available. The survey-based approaches found in the literature have one or more of the following drawbacks: (i) low response rate; (ii) poor international representation; (iii) slow turnaround time; (iv) little scale-up potential; (v) they are one-off studies, not easily allowing for follow-up(s).

We here present a survey-based approach to assessment of scientific community opinion designed to achieve a high response rate, include a broad and diverse set of scientists, allow for rapid implementation, be amenable to significant scale-up, and with the potential to be repeatable indefinitely.

The motivation for developing a new survey methodology with these virtues certainly isn’t to replace other methodologies, of which there are many. For example, there is still plenty of space for Delphi methods [9, 10], literature analysis techniques [1], and more targeted surveys of a relatively small number of specialists [2, 5]. Each approach has its advantages and disadvantages, and the choice of methodology will depend on precisely what one hopes to show. For example, if one wishes to ascertain scientific opinion regarding a specific and carefully constructed statement, a literature-based approach may perform poorly: that *exact* statement may not even exist in the literature. At the same time, one may wish to know what scientists think *right now*, not across literature spanning several years. In addition, there are options for triangulation: in some cases, it may be possible to demonstrate a strong consensus on a certain topic, no matter what method one employs. In other cases, it may be revealing to see that a strong consensus exists according to most scientific opinion assessment methods, but not all.

We argue that our newly developed method constitutes a valuable complement to currently available methods for ascertaining the degree of scientific agreement both across scientific fields and within particular subcommunities of scientists (as appropriate). The method can be applied to numerous scientific issues, potentially informing policy decisions and public understanding across diverse fields, and on a global scale. In particular, we highlight recent international expansion of the network, to incorporate *c*.50,000 scientists across 80 institutions. The method can tease out potential differences of opinion among scientists located in different regions, thereby allowing for sensitivity to local conditions that might be especially relevant to policy issues.

For the sake of this proof-of-concept study we chose the following statement, (S):

(S): *Science has put it beyond reasonable doubt that COVID-19 is caused by a virus*.

Many other statements could have been chosen. For present purposes we merely wish to set a baseline regarding what scientific consensus looks like on our particular methodology. Whilst the survey results are not uninteresting, our priority is to develop a novel survey method with important virtues. The key methodological innovations are:

(i) Each scientist receives a personal, one-to-one survey invitation email from somebody inside their own institution.
(ii) The survey invitation email, and the survey itself, are both maximally concise.

In June 2023, a group of 30 philosophers and social scientists sent survey invitation emails to 20,085 scientists across 30 institutions in 12 countries, gathering 6,807 Likert scale responses. In nearly all cases, survey invitation emails were sent internally, by the local coordinator (or ‘spoke rep’) at each of the 30 participating institutions, targeting academic scientists at that institution with their email addresses in the public domain. An example of the survey invitation email is presented in the next section, together with the survey platform itself. Details of the methodology, and its underlying justification, are also presented in the next section. Readers primarily interested in the survey results, and their interpretation, may wish to skip forward to the ‘Results and discussion’ section.

## Method

**Fig 1:**
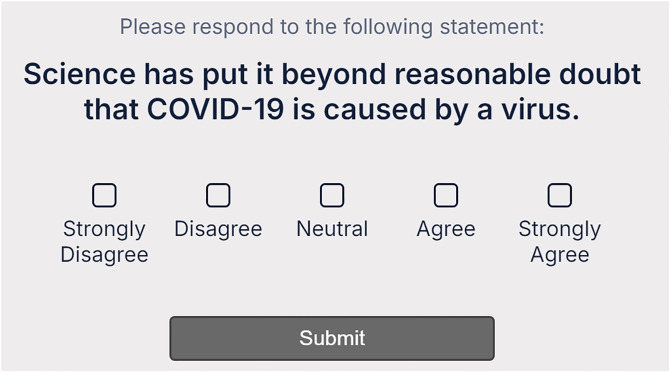
The ‘10 second survey’ platform used.

This study was pre-registered on 2^nd^ September 2022 using the Open Science Framework, with the title ‘New method of measurement for strength of scientific consensus regarding a specific statement’; see https://osf.io/dgq4k. However, we departed from the registered plan in certain ways:

(i) Research software engineers at Durham University, UK, created a bespoke surveying platform (Fig 1). This was a vast improvement on the original vision, as described in the pre-registration.
(ii) We scaled up to c.20k scientists at 30 institutions. This was done to improve the scale of the project, making it a more effective test of the methodology, but also because scaling up was relatively easy with the bespoke surveying platform in place.
(iii) We chose a different survey statement. This was done on the grounds that a more serious survey, concerning a more controversial and politically sensitive statement, should not be used until the methodology has been improved following a series of iterations.

Once the project concluded, we also used the Open Science Framework to create a permanent record of our data, code, and figures generated [11].

Making use of existing academic relationships, we established a network of 30 participating academic institutions. This included finding 30 local coordinators, or ‘spoke reps’, who would send out emails internally, at each institution, using a mail merge application. The majority of the spoke reps were philosophers of science, broadly construed, already known to each other through collaborations and conferences. A few were social scientists, or philosophers of science who collaborate with social scientists and are no strangers to empirical methods. Early on, we judged that the key thing for boosting response rate was that an academic internal to the scientist’s institution invited them to participate, regardless of the specialization of that academic. At two institutions (Leeds and UCI) we allowed doctoral students to send the survey invitation emails, as something of an experiment, to see if this had a significant impact on response rate.

During the period 10^th^ December 2022 through 15^th^ April 2023, five individuals based at the University of Durham prepared spreadsheets of scientists’ names and email addresses for the 30 institutions, finding email addresses in the public domain. It was necessary that (i) scientists were in no way cherrypicked, and (ii) clear guidelines were in place for identifying ‘scientists’. No definition of ‘scientist’ was required; instead, uncontroversial examples of ‘scientists’ were identified using sufficiency criteria. Scientists needed to be clearly affiliated with a university department, institution, or centre, and have a PhD in a relevant field, or equivalent qualification (see below). Scientists working in industry were not included, since they don’t tend to have handy identifying criteria, or email addresses in the public domain, and for ethical reasons we could only send our ‘unsolicited’ survey invitation emails to scientists with email addresses in the public domain.

Potential participants were grouped into five broad fields: Physics; Chemistry; Biology; Earth Sciences; Health Sciences. Most scientists belonged to a department which could easily be assigned to one of these broad fields–for example, membership of a physics, chemistry, or biology (or biological sciences) department. Those assigned to ‘earth sciences’ were found in centres, departments, or schools, with a wide range of names; these included the ‘School of Earth and Environment’ at the University of Leeds, the ‘Department of Earth System Science’ at UCI, the ‘School of Geography, Earth and Environmental Sciences’ at the University of Birmingham, the ‘Centre for Earth, Ocean and Atmospheric Sciences’ at the University of Hyderabad, the ‘Department of Earth, Atmospheric, and Planetary Sciences’ at Purdue University, and the ‘Department of Geology’ at the University of Pretoria. In nearly all cases it was straightforward to assign a scientist based in one of these centres, departments, or schools to the ‘earth sciences’ field. We opted not to include academics based in geography departments, even though there is no sharp distinction between geologists and physical geographers; natural scientists were our focus in this particular study, and many academics based in geography departments are best described as social scientists. Our intention was never to include all relevant individuals at a certain institution; rather, it was our intention to ensure that all those that *were* included met our criteria.

The field of ‘Health Sciences’ was another complex field. Scientists placed in this category came from academic centres, institutes, departments, and schools with a wide range of names. These included the ‘Department of Public Health and Primary Care’ at the University of Cambridge, the ‘Sue and Bill Gross Stem Cell Research Centre’ at UCI, the ‘School of Population and Public Health’ at UBC, and the ‘School of Dentistry’ at the University of Leeds. At the University of Exeter, within the Faculty of Health and Life Sciences, we included the ‘Department of Clinical and Biomedical Sciences’, but not the ‘Department of Psychology’. This was a judgement call based on the fact that members of staff in psychology departments often are not working in ‘health sciences’, and have varied backgrounds, with some having PhDs in Philosophy, for example. To save the time of painstakingly examining the credentials of every member of a psychology department, we simply left them out of this study. As this project develops and progresses (see ‘Outlook’) such choices will certainly be revisited.

**Fig 2:**
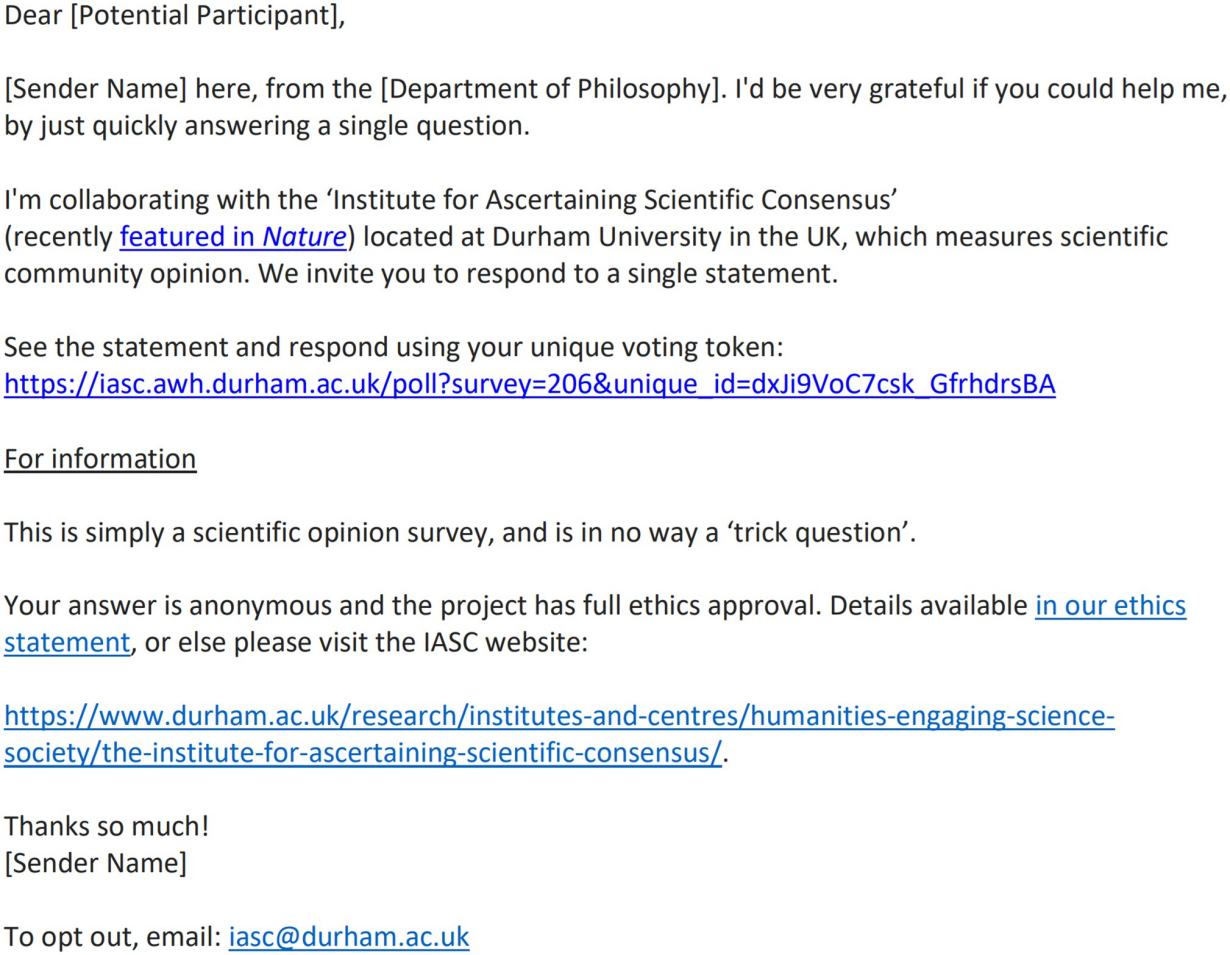
One example of the survey invitation email; adjustments across institutions were made, as needed, to account for local norms.

Each potential participant’s broad field of expertise was encoded in their unique voting token (see Fig 2), so that results could be carved up accordingly and analysed. Some critics might prefer *narrow* fields of specialization (such as in [2] and [5]), on the grounds that one does better to survey ‘true experts’ as opposed to scientists merely working in the same broad field. However, the notion of ‘true expert’ has been used in the past to exclude important voices from a scientific conversation ([12], see especially Chapter 5). Moreover, it is controversial to suppose that the only worthwhile opinions are those *directly* informed by ‘first order’ scientific evidence; arguably, scientists use ‘higher order’ evidence all the time ([13], pp. 135–53). In addition, in some cases, focusing on highly relevant specialists will mean that one’s final number of participants is very small, and this can lead to problems such as self-selection effects, or echo-chamber effects. The larger the participant number, the more likely it is that one’s cohort will incorporate diverse perspectives, and the harder it is for a sceptic to mount an accusation of bias or cherry-picking. At the same time, we emphasise that our methodology is compatible with future adjustments where scientists are categorised more narrowly into sub-fields, e.g. ‘Astrophysics’. Consider, for example, the survey undertaken as part of the Leverhulme-funded project ‘Exploring Uncertainty and Risk in Contemporary Astrobiology’, or ‘EURiCA’ (a collaboration between philosophers and scientists based at the universities of Durham and Edinburgh in the UK). During February 2024, as part of this project, a decision was made to survey the *astrobiology* community (broadly construed), with non-astrobiologist scientists included merely for the sake of comparison with the target group. The same methodology was utilized, with the exception that the PI of the ‘EURiCA’ project (Vickers) sent all of the emails personally. We note that the appropriateness of asking experts more broadly, or more narrowly, will depend on how ‘hybridized’ the focus statement is, in the sense of Ballantyne’s ‘hybridized questions’ [14].

In a few cases, an institution’s participants came from only some of the five fields. For example, the University of Exeter, in the UK, doesn’t have a Chemistry Department. A few professional chemists do work at Exeter, but we did *not* include them since doing so invited problems vis-a-vis anonymity of participants. If all scientists in a particular field (e.g. Chemistry) at a particular institution (e.g. Exeter) vote, and vote the same way, then it becomes possible to say how a particular scientist voted. We made this very unlikely, by ensuring that no fewer than ten scientists occupied any such field+institution category. If any field+institution category were going to be occupied by fewer than ten scientists, we excluded the category entirely. Thus, for example, Exeter participants came from ‘Physics’, ‘Biology’, ‘Earth Sciences’, and ‘Health Sciences’. Similarly, Lancaster lacked participants from ‘Biology’, NYCU Taiwan lacked participants from ‘Earth Sciences’, Stockholm lacked participants from ‘Health Sciences’, and Amsterdam lacked participants from both ‘Biology’ and ‘Health Sciences’. If, in the future, we *were* to use narrower specialization categories, e.g. ‘Astrophysics’ instead of ‘Physics’, this problem of ensuring anonymity would amplify. So too, acquiring additional data such as seniority of participant would threaten anonymity. It has been widely documented that de-anonymization is a serious threat [15–17].

Many of the targeted participants were easy to select, e.g. a Physicist working in a Physics Department, with a PhD in Physics. Occasionally an EngD compensated for not having a PhD. The ‘Health Sciences’ category was more challenging, and here we allowed for participants with MD, PsyD, DVM, or PharmD qualification. Sometimes the title ‘Honorary Professor’ helped to indicate that an individual was a respected scientist, even though they’d had an unusual career trajectory. In a small handful of cases an individual was included without the expected qualifications, but with every other possible indicator, e.g. a distinguished career and long list of relevant publications in respected scientific journals. To emphasise, only uncontroversial cases of ‘scientists’ were used; if there was any doubt regarding an individual’s academic status, they were not included. Some judgement calls had to be made; however, with very large numbers of scientists involved, and a small number of borderline cases, results remain robust across different possible judgement calls.

In parallel, Research Software Engineers (RSEs) at Durham prepared bespoke surveying software and the survey platform (Fig 1). The completed spreadsheets could be entered into the platform to generate unique voting tokens for every potential participant, ensuring that only those targeted could vote, and each person could only vote once. The voting tokens also tagged votes with institution and discipline of voter, but no other information. Raw voting data thus took the form “Durham, Physicist, Strongly Agree”, “Hyderabad, Earth Scientist, Disagree”, etc. Whilst the survey was open, it was possible for those with access to the server (i.e. the software engineers) to see which targeted scientists had voted, since they could see which tokens had been used. When the survey closed (after four weeks), all tokens were deleted from the server, and it was then impossible for anyone to ascertain even *if* a given targeted scientist had voted. To be clear, no ‘field+institution’ category had a 100% response rate; if it had, we *would* be able to judge that a targeted participant in the category had voted.

Following standard beta-testing with a Beta Testing Group of 50 scientists, initial invitations were sent out during June 2023, with a single reminder email sent out two weeks later, and the entire survey closed after four weeks. In just one case–UNAM–the reminder email wasn’t sent. In three other cases, due to technical or ethical challenges and last-minute complications, the local spoke rep was unable to send the survey invitation email, the reminder email, or both. In these cases, the project lead (Vickers) performed the mail merge externally, from a Durham University email account. It is unclear whether or not this had a serious effect on the resultant response rate, since several factors affect response rate.

Not only was the survey itself maximally short (Fig 1), the survey invitation email was also maximally short (see Fig 2), while still including all necessary information. It was imperative that the email itself be maximally short, since this was the first thing a potential participant would see, and there was no point having a super-short survey if the scientist never clicked on the link to reach the survey. Sometimes a scientist will see a long email, and quickly judge that even just to read the email is too big an ask of their time.

The bespoke surveying software and platform have already been published on Zenodo [18]. We conducted this pilot project on a very limited budget, and thus the survey software is limited in various respects. For example, the raw data for each institution had to be downloaded, pieced together, and processed, whereas in a future iteration a huge amount of data processing would happen automatically, including generation of an ‘initial report’. Similarly, opt-outs had to be processed manually. It would also be interesting to acquire data on the percentage of potential participants that *did* click the link in the survey invitation email, but did *not* vote, or only voted much later (following the reminder email). As discussed in the next section, participants voting in the second wave tended to disagree more than participants voting in the first wave. In future iterations of the method, we could test whether disagreement votes in the second wave correlate with opening the survey, but not voting, during the first wave.

We next turn to sampling bias concerns. Sampling happens twice: once from the entire population, and again when scientists themselves choose whether or not to participate. In both cases, sampling bias could occur. Is this a concern?

In the first case (kind 1), if (for example) all scientists within China were to disagree, then our survey results would *not* reflect international scientific community opinion; we’d get a biased result, and we wouldn’t know it, because we have no data from inside China.

In the second case (kind 2), if (for example) there were a strong correlation between *strongly disagreeing* and *not participating*, then we’d get a biased result. And once again we wouldn’t know it, because we don’t have any data from those that didn’t participate.

We respond as follows:

(i) Our survey is one of the most balanced scientific community surveys that has ever been done, involving a greater number of scientists, across more countries. Ideally we’d include *even more* scientists across *even more* countries, and in fact there are concrete plans for this (see ‘Outlook’). An additional point is that the diversity is greater than it initially appears. For example, our set of targeted Oxford University scientists includes scientists from more than 30 different countries, when judged by country of academic formation (typically BSc, MSc, and doctoral study). We stress, however, that since votes are entirely anonymous, we cannot determine which of the targeted Oxford University scientists participated.
(ii) We achieved a high response rate relative to other survey-based methodologies, and the higher the response rate the lower the worry about ‘kind 2’ sampling bias. Acquiring data on gender and age (see e.g. [2]) doesn’t help much with such bias, since the primary bias could easily come in elsewhere, such as with political ideology [19, 20].

Apart from these sampling bias concerns, it has been suggested that scientists in a certain region might be somehow biased, simply in virtue of being based in that region. This is plausible; Vickers ([12], Chapter 5, Section 2) discusses how, in the 1930s, 40s, and 50s, scientists in certain countries (including the US, UK, and Australia) were biased towards dismissing ‘continental drift’ as a hopeless idea, whilst other countries (such as South Africa) were more open-minded. This had to do with the contrasting scientific cultures in those countries. Crucially, the survey approach being proposed in the current paper would serve the purpose of revealing such differences, if they exist. Data on differences in scientific opinion across countries/regions is valuable data to have. If differences are revealed, one can then conduct an investigation to determine whether those differences are indeed due to problematic biases, or echo-chambers, or are simply reasonable differences of professional judgement. We see our role as the first step in this process–collecting the initial data, to reveal a possible bias, which can then be investigated directly.

Finally a note on ethical approval. This project has been through a comprehensive ethical approval at Durham University, UK, equivalent to IRB. A full Data Protection Impact Assessment (DPIA) has been completed, including a Legitimate Interests Assessment (LIA). The assessment result for this project is ’low risk’. Detailed documentation of (i) ethical approval, (ii) DPIA, and (iii) LIA, is included as Supporting Information ‘Other’. We note here that the need for participants’ explicit prior consent was waived by the ethics committee. A full discussion is available in the Supporting Information ‘Other’. Just briefly we stress here that if participants did not consent, they could simply ignore or delete the survey invitation email. Clicking the survey link embedded in the email is equivalent to consenting for one’s opinion to be included, anonymously, as part of the survey. On the scale of many thousands of scientists it would be virtually impossible to acquire explicit consent from participants, without very heavily impacting the return rate, and thereby undermining the project. Thus another way must be found, and it is ethically sound to take a scientist’s decision to click the survey link in the invitation email as signalling their consent to their opinion being included in the survey. Scientists are able to permanently ‘opt out’ in two clicks.

## Results and discussion

We hypothesised that our method would either already achieve, or show clear future promise of achieving, five desirables. Three are directly tested by our study:

(i) high response rate;
(ii) responses from a broad and diverse set of scientists;
(iii) rapid turnaround of results.

Two further desirables are also plausible on this methodology, and merit discussion:

(iv) scale-up potential;
(v) minimal survey fatigue, allowing for significant repeatability.

As we set out to show, desirable (iv) falls out of the basic methodological setup. Desirable (v) is a plausible prediction, to be confirmed or disconfirmed by future studies.

**Fig 3:**
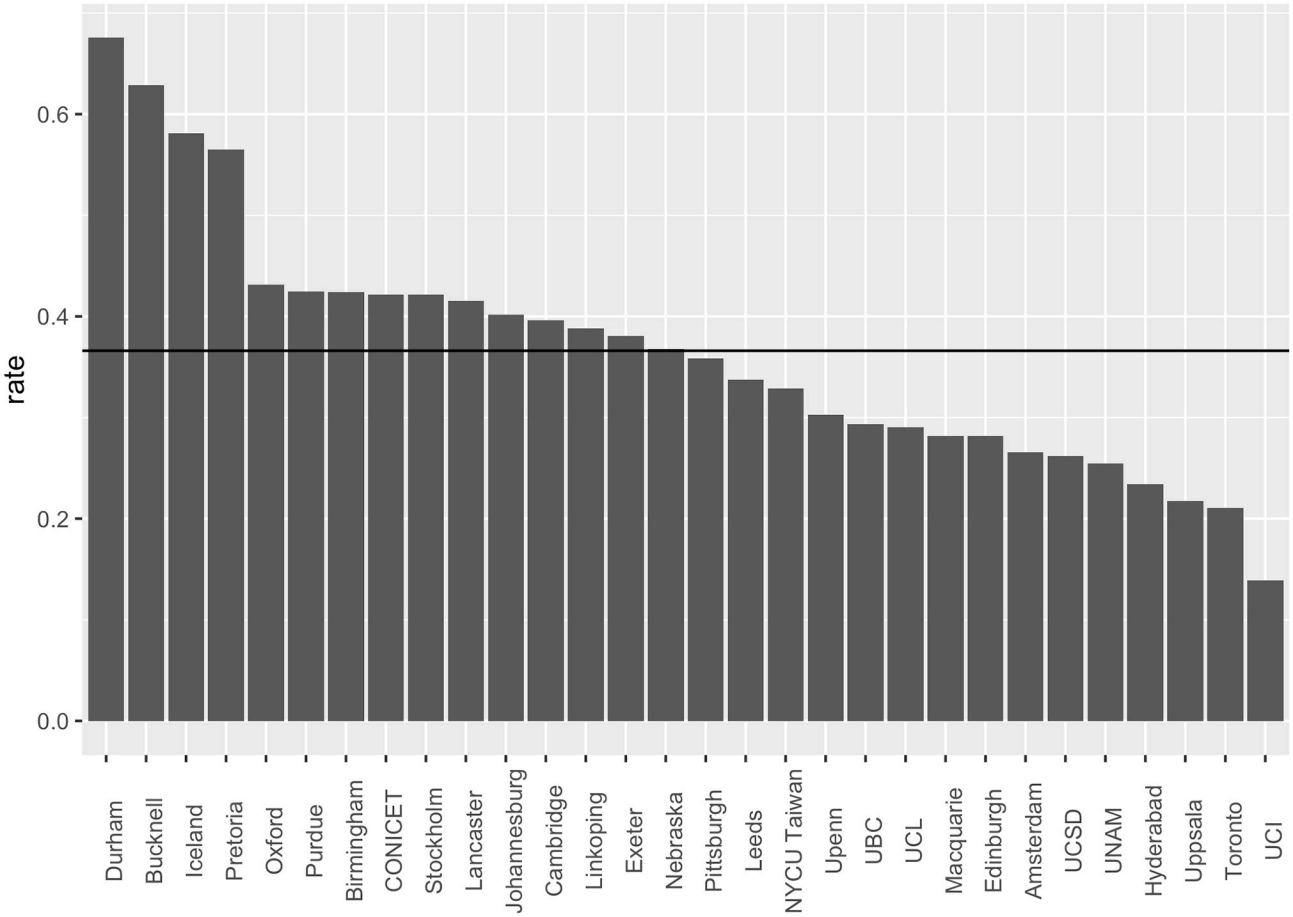
Survey response rates, by institution.

Turning to (i), the overall response rate was high, relatively speaking, at 33.9%. For comparison: the recent IRIS survey on research integrity [4] had a response rate of 7%, and the ‘Well-being in Science’ project led by Brandon Vaidyanathan worked hard to reach a 15% response rate [3]. Recently, the ‘Covid origin’ survey conducted by the Global Catastrophic Risk Institute achieved a response rate of 14.8% [5]. Even the leading climate change survey achieved only 25% response rate [2]. These numbers are typical; one would struggle to find any higher response rates, for this kind of work. Moreover, our response rate was relatively speaking *extremely* high (>55%) at four institutions (Fig 3). Very high response rates at a larger number of institutions will be possible in future surveys, given lessons learned and opportunity for methodological fine-tuning.

Regarding response rate, we also mention here a recent survey of the global astrobiology community. This was conducted as part of the aforementioned Leverhulme-funded project ‘Exploring Uncertainty and Risk in Contemporary Astrobiology’, or ‘EURiCA’. Disregarding ‘undeliverables’ (i.e. those emails that were sent but that didn’t reach an inbox), 1,176 astrobiologists were emailed on 10^th^ February 2024, with 357 responses received in the first 72 hours, a response rate of 30.4%. After two weeks the survey was closed, with a final response rate of 44.6%. This survey used the same novel survey method, with the exception that the PI of the EURiCA project (Vickers) sent all of the survey invitation emails personally.

Breaking response rates down by field of specialization, Physics has the best response rate, with Biology and Earth Sciences very close behind. The numbers are: Physics 38.33%; Biology 38.18%; Earth Sciences 36.97%; Chemistry 34.30%; Health Sciences 28.06%. The significantly lower response rate for ‘Health Sciences’ (statistical test: chi-squared *χ*^2^ (1, *N* = 6807) ≈ 112; *ρ*<0.001), as compared with all other scientists combined (one degree of freedom), is no doubt due to the fact that many such scientists are part-time clinical practitioners, for example working in the NHS. Such scientists are especially likely to simply delete an unsolicited request to complete a survey, however short. We also have reason to believe that such scientists receive survey requests even more often than other scientists, and are thus experiencing ‘survey fatigue’. But we stress that a 28% response rate is still good, relatively speaking.

**Fig 4:**
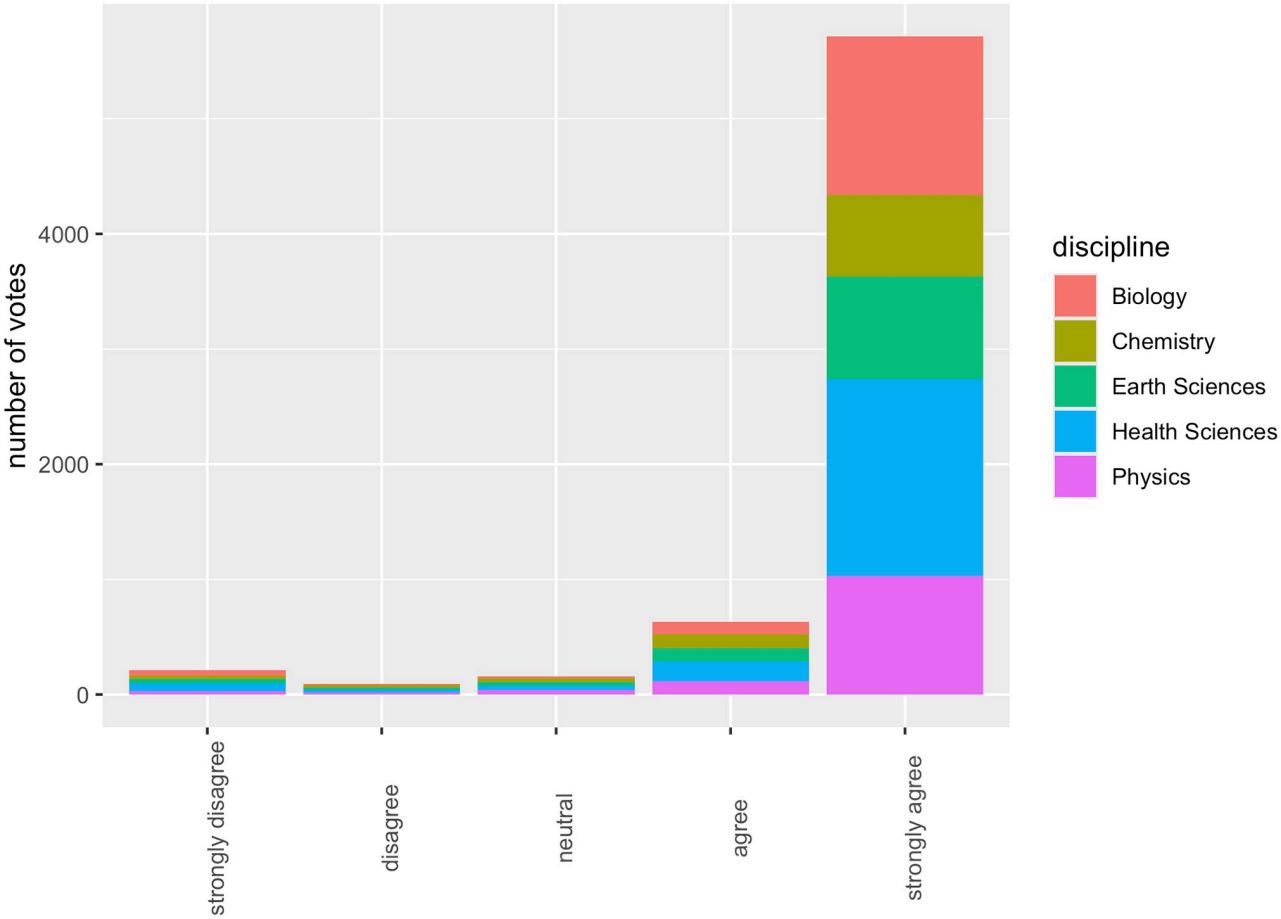
Likert scale survey results.

Turning to (ii), we collated responses from a reasonably broad and diverse set of scientists, with 6,807 participants across 30 institutions in 12 countries. Responses were spread across five broad scientific fields: Physics, Chemistry, Biology, Earth Sciences, and Health Sciences, and we take this opportunity to display our survey results (Fig 4), although we stress that these results are of secondary interest, given that our primary aim is to develop a novel methodology.

**Fig 5:**
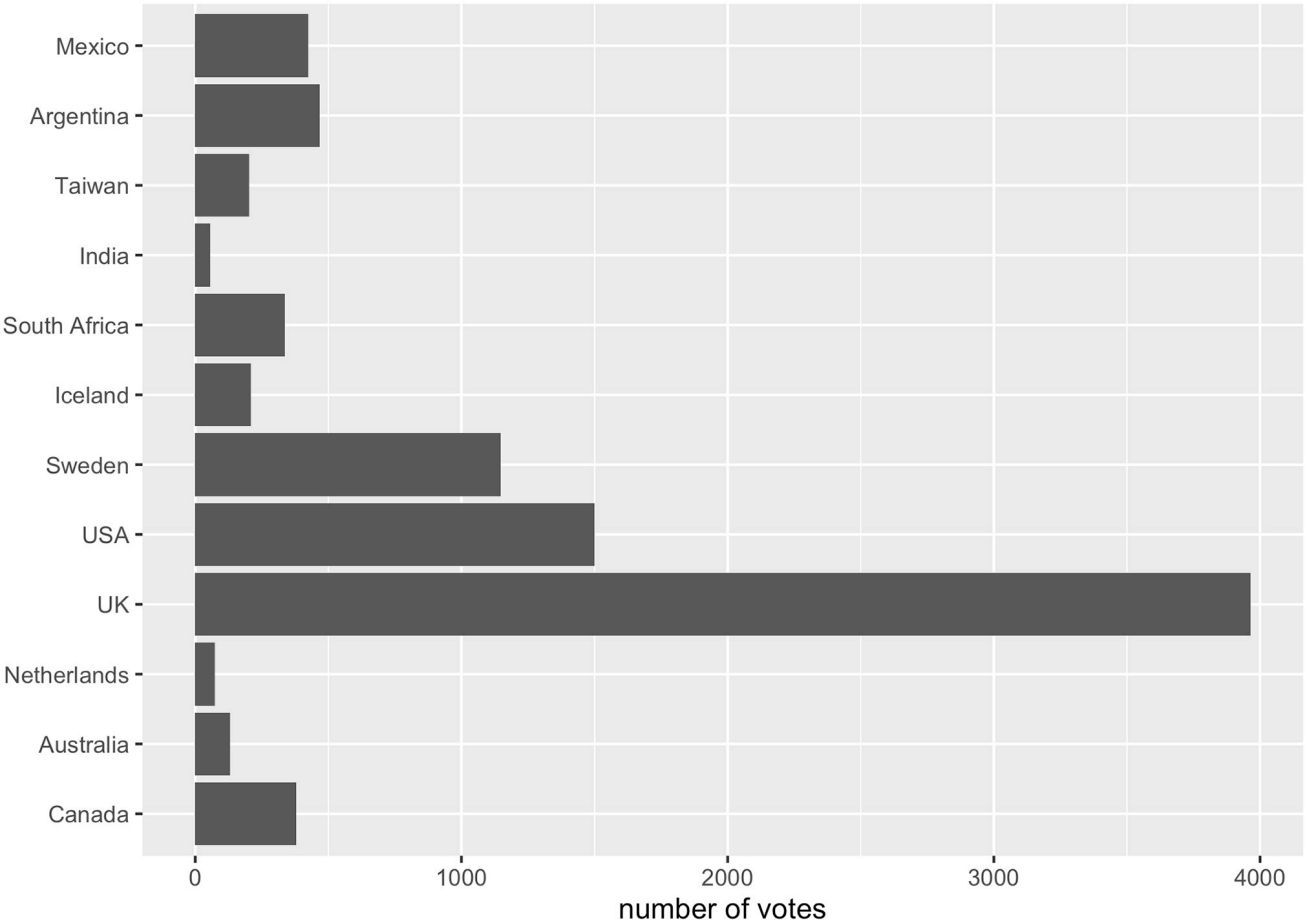
International vote distribution.

We were also able to collect data from multiple countries (Fig 5). We note that the international diversity of our response base, though falling far short of our ambition (see ‘Outlook’), is still an improvement on the typical state of the art (e.g. [2]). In surveys of scientific opinion to date, little attempt has been made to achieve results that are internationally representative. In one sense Ackerman et al. ([5]) is an exception, since global opinion is explicitly sought. However, for a ‘global’ survey their participant number N = 168 is extremely small, allowing each country involved a very limited voice. To compare: the recent expansion of our network to *c*.50,000 scientists at 80 institutions (see ‘Outlook’) shows what is achievable on our particular methodology vis-à-vis both international representation and scale-up potential.

**Fig 6:**
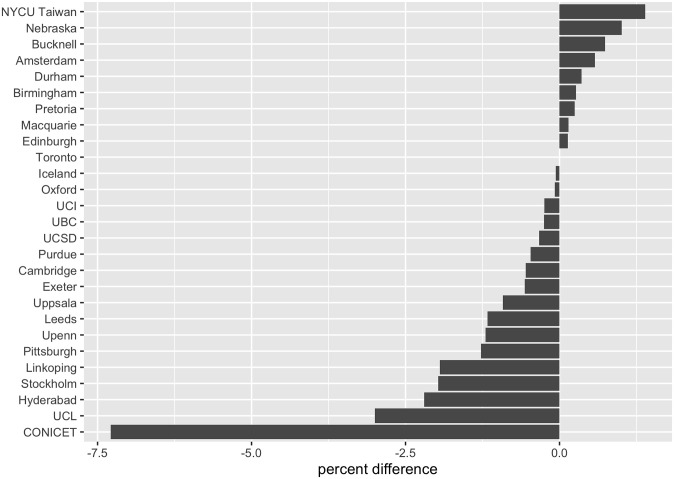
Agreement adjustment following the reminder email. (Data collected across 27 of the participating institutions).

Turning now to (iii), we achieved a reasonably rapid turnaround of results, with promise for very rapid turnaround in the future. Invitations to participate were initially sent mid-June 2023, with final results ready mid-July 2023, amounting to a turnaround of one month. We now ask: was the final agreement result essentially in hand long before the survey closed? Did the survey need to stay open for one month? Half-way through the survey, following 4,505 responses, overall agreement stood at 95.2%. Following an additional 2,302 votes in the second half, overall agreement stood at 93.2% (95% Confidence Interval *CI* = 0.932 ± 0.006). This constitutes a small but statistically significant dip in overall agreement (statistical test: chi-squared *χ*^2^(1, *N* = 6807) = 9.63; *ρ* = 0.002), although interestingly we found variation across institutions (Fig 6).

It cannot be inferred that the final agreement result was already in hand long before the survey closed, given that the agreement percentage dipped by 2% in the second wave (following the reminder email). Whether a reliable pattern of agreement dipping would emerge following many surveys remains to be seen. Consider the previously mentioned ‘EURiCA’ survey of astrobiologists which took place in February 2024. The overall agreement dipped during the survey as follows: 89.7% agreement after 48hrs, 88.8% after 72hrs, 87.5% at the end of Day 8, 86.6% at the end of Day 9 (following the reminder email), with a final agreement result of 86.5%. This gives a 2.3% dip in overall agreement between 72hrs and the final result. Thus, a good estimate of the final result might be possible after just 72hrs, but, potentially, with the necessity that one factors in a dipping phenomenon vis-à-vis overall agreement. The noted dipping phenomenon adds to studies exploring response time and decision making in a range of different settings [21–23].

One may ask under what circumstances a rapid turnaround of results, such as 72hrs, might be beneficial. Stegenga ([24]) discusses cases of ‘fast science’, where science must move quickly to respond to a ‘supreme emergency’. The COVID-19 pandemic was one such ‘supreme emergency’. A more common example concerns the importance of rapid turnaround in the context of assessing admissibility of scientific evidence in judicial proceedings. For example, scientific consensus is relevant to various standards of the admissibility of scientific evidence which apply throughout the US, including the Frye standard (and Kelly-Frye standard), and the Daubert standard. The Frye standard explicitly references “general acceptance” by the scientific community. The Daubert standard incorporates some elements of Frye; the evidence must rest on a “reliable foundation”, and general acceptance in the scientific community is relevant to assessing whether this is the case. The rapid turnaround of our approach could be especially helpful in this context.

Leaving discussion of scale-up potential for a later section (‘Outlook’), we turn next to repeatability. As already noted, survey fatigue will be most accurately assessed once the same set of scientists have been surveyed several times. Initial indications are somewhat mixed. Our overall opt-out rate was very low, at 0.3%, which is important for repeatability, since every ‘opt-out’ needs replacing with an alternative if one is to maintain one’s stock of potential participants. The opt-out rate for the ‘EURiCA’ survey of astrobiologists was less than 0.2% (two individuals).

**Fig 7:**
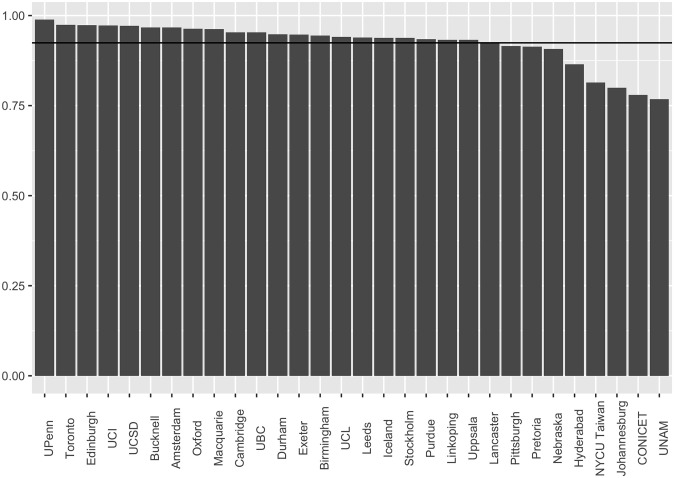
Survey results for percentage of agree or strongly agree, by institution.

An additional data-point comes from our re-survey of CONICET (Argentina’s leading public research institution) during December 2023-January 2024. This follow-up was conducted in light of the fact that CONICET’s response to the initial survey was an outlier, with a relatively low agreement score of 78% (see Fig 7). Due to budget constraints, we used the same stock of scientist names/email addresses for the follow-up survey, even though it was now one academic year out of date; this was bound to harm the response rate, for example because we included postdocs, who come and go quite regularly. From a single email we received 143 responses from 577 emails, which is a 24.8% response rate (no reminder email was sent). In the June-July 2023 survey, we received 165 responses from CONICET, from the initial email. Thus we see here some drop-off in the response rate, but this could potentially be compensated by updating the spreadsheet at the start of each academic year. It can also be more-than-compensated by introducing new institutions to the network as and when needed, something that is demonstrably easy to do (see ‘Outlook’).

It might be claimed that whilst our approach already fares quite well, and could perform very well, with regard to issues (i)-(v), it performs poorly on the most important issue: veridical representation of (relevant) scientific opinion. The overall agreement percentage was 93.2% (95% Confidence Interval *CI* = 0.932 ± 0.006), which might seem uncomfortably low for a statement such as (S). For example, it may appear low compared with other results in the literature, such as results of 97% agreement or higher regarding human-caused global warming [1, 2, 25, 26]. In addition, some of our institutions returned results that seem very low, e.g. at or below 80% (see Fig 7).

**Fig 8:**
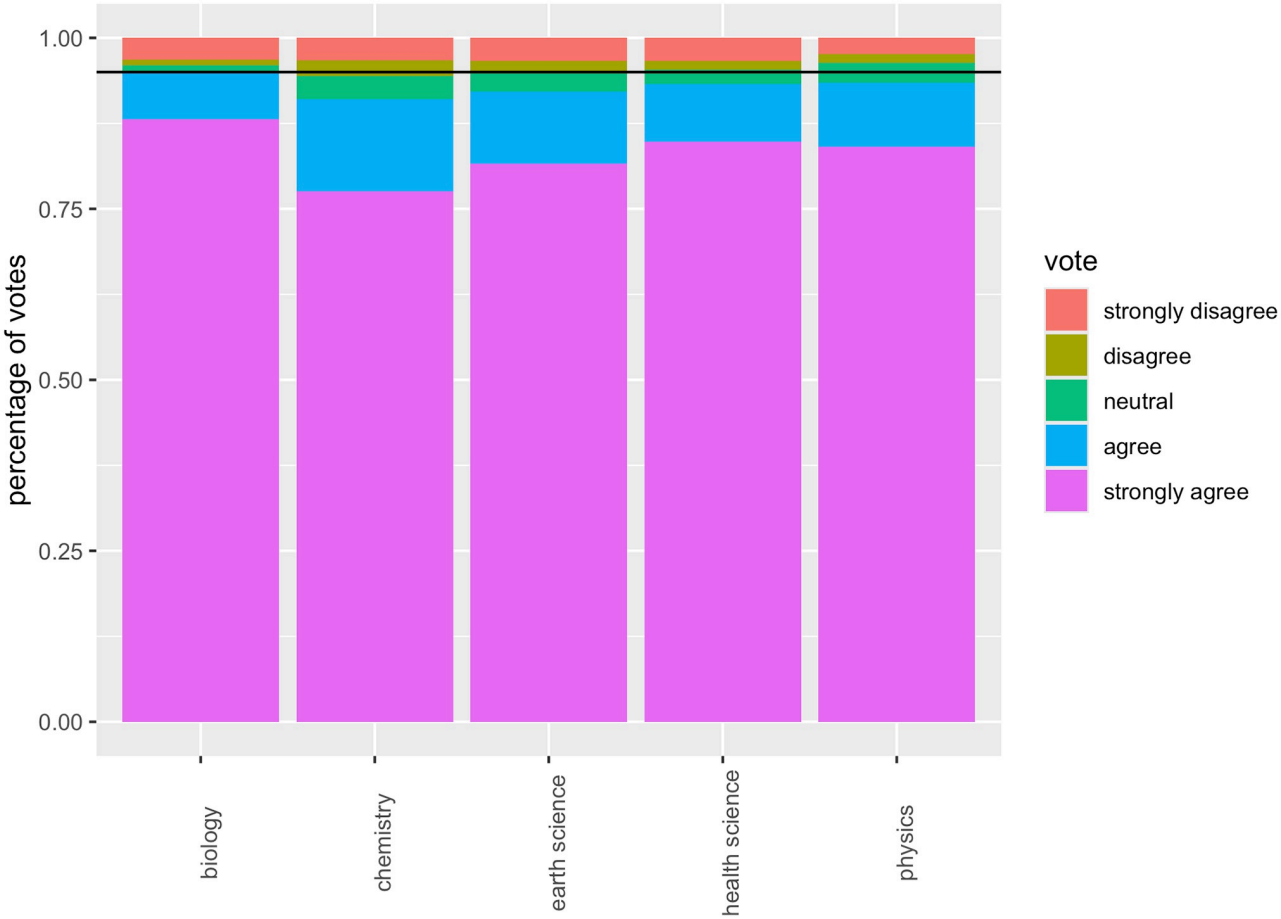
Results by field.

It might be suggested that the low overall agreement score of 93.2% has to do with the fact that we surveyed across Physics, Chemistry, Biology, Earth Sciences, and Health Sciences, thus including responses from scientists who have no expertise whatsoever when it comes to COVID-19. However, for a high-profile issue, such as the (basic) cause of COVID-19, field of expertise is relatively unimportant according to our results (see Fig 8). One cannot infer that the reason the agreement score was low was because we included physicists (for example) in a survey about Covid; the overall agreement percentage for physicists was 93.5%.

Regarding the overall agreement score of 93.2%, we stress that in addition to basic disagreement with statement (S), several explanations for non-agreement votes present themselves. (We stress that only some of these can serve to explain the relatively low agreement score, since some will be present in any survey-based approach).

1. Participants choose ‘neutral’, a response that is given for different reasons, including as a way to ‘hide’ non-response ([27]; see also [28]).
2. Participants hold to the philosophy that nothing in science should be ‘beyond reasonable doubt’, and we need to keep an open mind about everything, including the most obvious ‘facts’. This is particularly likely in some scientific circles, especially given the ongoing influence of Karl Popper.
3. Participants feel that (S) problematically simplifies a complex reality. To illustrate: one participant explained that Covid was *triggered* by a virus, but was caused by many things, including lifestyle and comorbidities.
4. Participants enjoy being contrarian; they hold to a philosophy where, allegedly, challenging widely accepted ideas, including ‘facts’, is crucial for scientific progress. An interesting example concerns American geologist Arthur Mirsky, who, in the context of the continental drift debate, stated, “I got the urge to sound more hardline … I needled them [‘drifters’] with my apparent hard-line opposition rather than my actually more moderate, questioning view.” ([29], p. 372).
5. Participants are inattentive or mischievous. See e.g. [30], p. 3: “Inattentive and mischievous respondents (collectively referred to in this report as ‘problematic respondents’) can bias the results of surveys by dramatically inflating point estimates and creating illusory associations.” (See also [31]).
6. Participants were slightly nudged towards selecting the first response option listed (‘Strongly Disagree’), simply because it was first. (See for example [32].)
7. Unexpected local contingencies result in unusually low agreement scores at a small handful of institutions (see Fig 7), bringing the overall average down.

Some may prefer to see action taken to minimise at least some of these factors, in order to increase the consensus score closer to 100%. In some cases, a result perceived as ‘low’ might be considered politically problematic, a consideration that could prompt researchers to design surveys that achieve very high consensus results (cf. [26]). For example, if one really *wants* to get a very high consensus result for anthropogenic climate change, one could ask only IPCC authors. One could also remove the clause ‘Science has put it beyond reasonable doubt’, since this clearly raises the bar.

However, lower agreement results need not mean that the scientific claim is less credible [33]. In addition, if our purpose is to set a baseline, there are good reasons not to minimise factors 1–7 and instead allow for their influence on the agreement rate. After all, these factors are likely to also be present in other surveys of scientific opinion, including future instances of our own approach. Thus, any interpretation of these other results using our results here as a baseline would be most reliable if our baseline results reflect these factors as well. For example, if our baseline is approximately 93% with factors 1–7 not minimised, then a similar survey on anthropogenic climate change (say), including the clause ‘Science has put it beyond reasonable doubt’, shouldn’t be expected to get above 93% in order to count as a solid consensus. At least some of the considerations 1–7 noted above can be considered as ‘noise’ [34, 35], some degree of which is to be expected in any survey of expert opinion. Making methodological adjustments to reduce the degree of noise from 4% to 3% (say) is unimportant if we are taking a baseline approach to assessing degree of scientific agreement.

It could still be claimed that the agreement results at UNAM and CONICET (see Fig 7) cry out for explanation, given their significant outlier status. Of course, it is possible that scientists in Mexico and Argentina are more sceptical than elsewhere and, if so, this is an interesting result and should be factored into the overall global agreement score. On the other hand, if scientists in these countries are *not* more sceptical than elsewhere, then these low results could be considered problematic, in which case it could be claimed that they should *not* be factored into the global agreement score.

In consultation with the spoke rep at CONICET we organised a follow-up survey in Argentina, to test our assumption that an unusual degree of scepticism amongst the scientists was *not* a plausible explanation of the low agreement score of 78%. In the new survey we chose.

### La evidencia científica nos muestra que el COVID-19 es causado por un virus

This translates to, “Scientific evidence shows us that COVID-19 is caused by a virus.” This is a useful creative translation of (S), which avoids the potentially problematic term ‘beyond reasonable doubt’ whilst playing a very similar role–it represents an achievable high-bar epistemic threshold, beyond ‘good reason to believe’, but falling short of ‘certainty’.

On 29^th^ December 2023 we emailed 577 CONICET scientists, receiving 143 responses. We received 127 ‘strongly agree’ responses, 15 ‘agree’ responses, and 1 neutral response; thus, the overall agreement score was above 99%. Whilst not definitive, this supports our hypothesis that scientists in Argentina are not unusually sceptical about the viral cause of Covid. Possible explanations of the prior, low agreement result of 78% are:

1. **Epistemic contextualism/higher bar.** Many scientists educated in the Argentine system take philosophy of science courses, where they encounter issues in epistemology. Furthermore, the Spoke Rep in Argentina introduced herself, in the survey invitation email, as a philosopher. Participants might have imagined that the question was not really about their scientific beliefs, but about their sensitivity to philosophical subtleties. Studies show that responses to questions can be affected by context, including location (e.g. inside/outside the philosophy classroom), and who asks the question [36, 37].
2. **Interpretation of ‘beyond reasonable doubt’.** To scientists in Mexico and Argentina, the phrase ‘beyond reasonable doubt’ can feel clumsy and awkward. Some scientists attempt to break it down word by word, resulting in an interpretation inconsistent with other countries, and different from that intended (cf. [38]). Generally speaking, survey results are often sensitive to issues of language and interpretation [39, 40].

Thus, one may worry that the initial CONICET results were an underestimate, and that, as a result, the global agreement result of 93.2% is too low. On the other hand, it is plausible that unexpected local contingencies will always affect large-scale international surveys conducted across multiple languages and cultures. If true, the baseline percentage for a strong scientific consensus ought not to be inflated by labelling certain unusually low results ‘problematic’.

**Fig 9:**
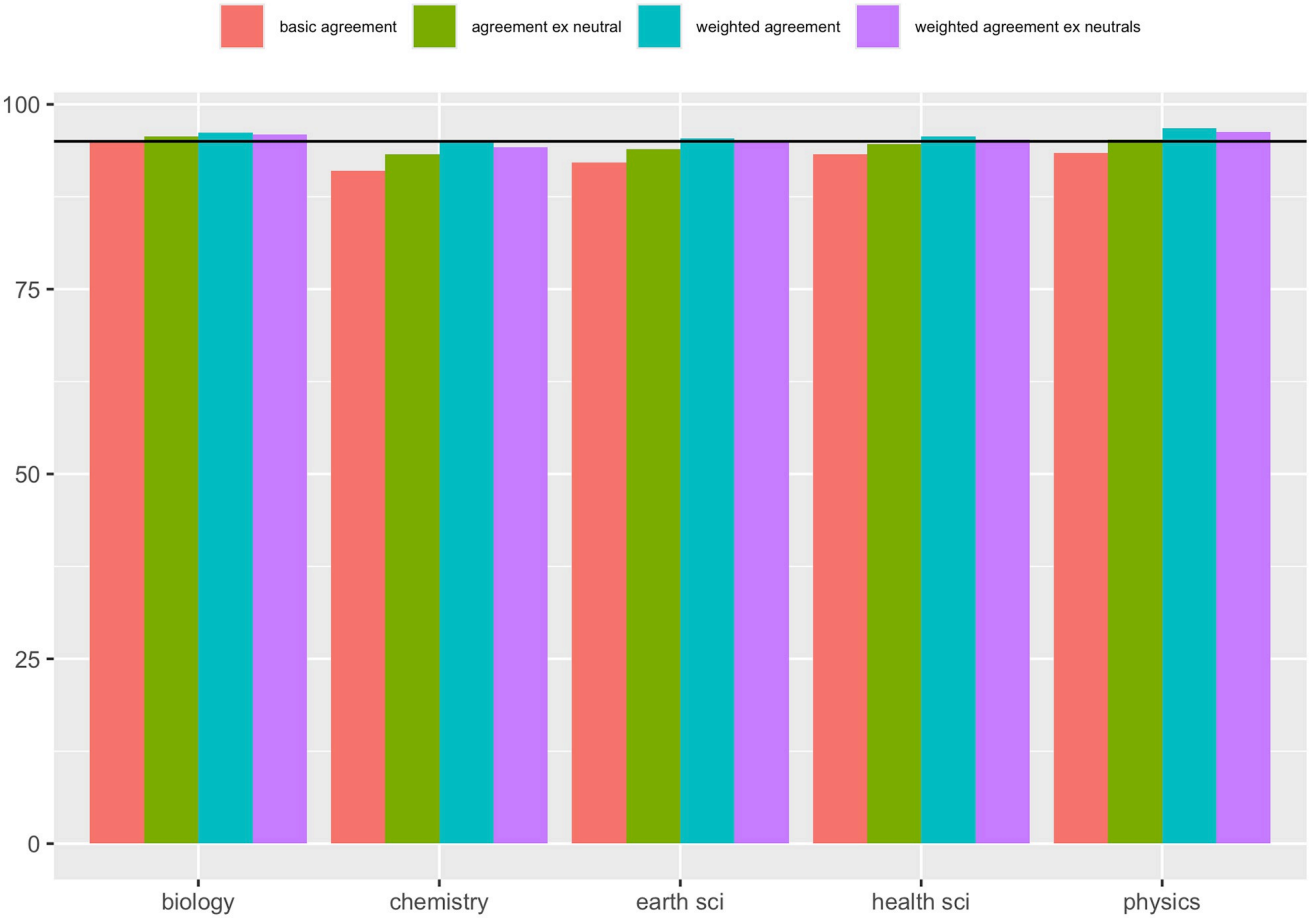
Illustration of how two decision points lead to four different ways to derive a measure of agreement from the data.

The focus on setting a baseline helps us to avoid various difficulties. For example, it is normal to present consensus results quantitatively, e.g. 97% (cf. [41]). But any method for measuring scientific opinion includes a large number of decision points, more than might be expected (cf. many-analyst studies, e.g. [42]). With two decision points, there are four different ways to reach a ‘consensus score’ from our survey results (see Fig 9).

For example, one could disregard neutral votes, perhaps on the grounds that many scientists choose this response because they don’t have an opinion, or don’t wish to offer an opinion, meaning that a ‘neutral’ response is comparable with choosing not to vote [27]. Additionally, one might want the consensus measurement to reflect the fact that the majority of votes were ‘strongly agree’ and not merely ‘agree’; otherwise, we get the same score regardless of whether we have a large majority of ‘agree’ responses, or a large majority of ‘strongly agree’ responses, and clearly these are two very different datasets vis-à-vis scientific community opinion. One option here is to double-weight ‘strongly’ responses (at both ends of the Likert scale).

Now if, for example, we ask, “Have health scientists reached a 95% agreement?”, the answer is unclear, since it depends on how you derive an agreement percentage from the raw data, and there isn’t a single correct way to do that. It would even be reasonable to respond “That’s a bad question”. But if one wishes to set a *baseline* which can be used to assess future survey results, it may not matter which choices one makes. The key thing is to ensure one takes the same approach as was taken in the baseline survey, so that results are not incommensurable, and a comparison can be made.

A final topic meriting discussion is ‘false balance’. False balance is a well-known problem in epistemology generally, and in scientific communication specifically [43]. The basic problem is that a consumer of information often sees far more balance across ideas than really exists. In an extreme case, the consumer sees ‘two sides to the story’, e.g. on an issue such as climate change [8].

But with our new methodology, the problem of ‘false balance’ may be overcome. Suppose we have 50,000 scientists in our network (see ‘Outlook’), and a 40% response rate is achieved, meaning that 20,000 votes are recorded. A 93% agreement here equates to 18,600 agreement votes, or *c*.1,400 non-agreement votes. Thus, some percentage of scientists are dissenters, potentially up to 7%, in an entire population of millions. Somebody wishing to dismiss the result might then amass many scientists who are dissenters. Suppose they amass 1,000 such dissenters. What would that show? Since the 93% consensus result already entails some non-negligible percentage of dissenting scientists, and since we are dealing with very large numbers of scientists, it is trivial that there would be 1,000 scientists who would disagree. There were more than 1,000 non-agreement votes *in the sample*, never mind when one turns to the entire population of scientists.

The exception might be if we are looking only at relevant scientists *narrowly* construed, since then we might *not* be dealing with large numbers of scientists, in which case even 500 scientists might be a significant number (depending on the overall number of scientists working in the field). If one actually can amass 500 dissenting scientists who are highly relevant experts, then that really might be significant, and the result should be welcomed. What one normally finds, however, is that in order to amass a potentially influential number of dissenting scientists, counterestablishment/anti-science groups have to interpret ‘scientist’ very broadly. Such is the case with the 1000+ ‘scientists’ disagreeing with the theory of evolution, as organised by the Discovery Institute: they are happy to include mathematicians, computer scientists, surgeons, civil engineers, and others–not exactly highly relevant experts (see dissentfromdarwin.org). In fact, if we interpret ‘scientist’ *this* broadly, there are at least 10M scientists in the world, and so 1,000 dissenting scientists is compatible with a 99.99% consensus. Indeed, were the Discovery Institute to amass 10,000 dissenting scientists–an *apparently* very significant number–that would still be compatible with a 99.9% consensus vis-à-vis the truth of evolution.

Another counterestablishment/anti-science organisation, Clintel, are behind a statement that “There is no climate emergency”, with nearly 2,000 signatures. The list of signatories can be found online, and the same story applies: even if they reach 10,000 signatures, that is compatible with a 99.9% consensus that there *is* a climate emergency.

A similar but more academically respectable approach can be seen in Hilbeck et al. [44], who wish to challenge the scientific consensus on GMO safety. They find 300+ researchers willing to stand opposed to the so-called ‘consensus’. But 300 researchers, whilst able to fill a large lecture theatre, is a tiny dissensus *percentage*, easily compatible with an extremely strong consensus. The authors refer to “strict qualification requirements” for signatories, but their signatories include non-scientists such as legal experts and ethicists. Thus the qualifying criteria are quite broad, and this makes 300 a very small percentage. These kinds of moves always focus on *numbers* of dissenting individuals, not a percentage score in a representative sample.

## Outlook

**Fig 10:**
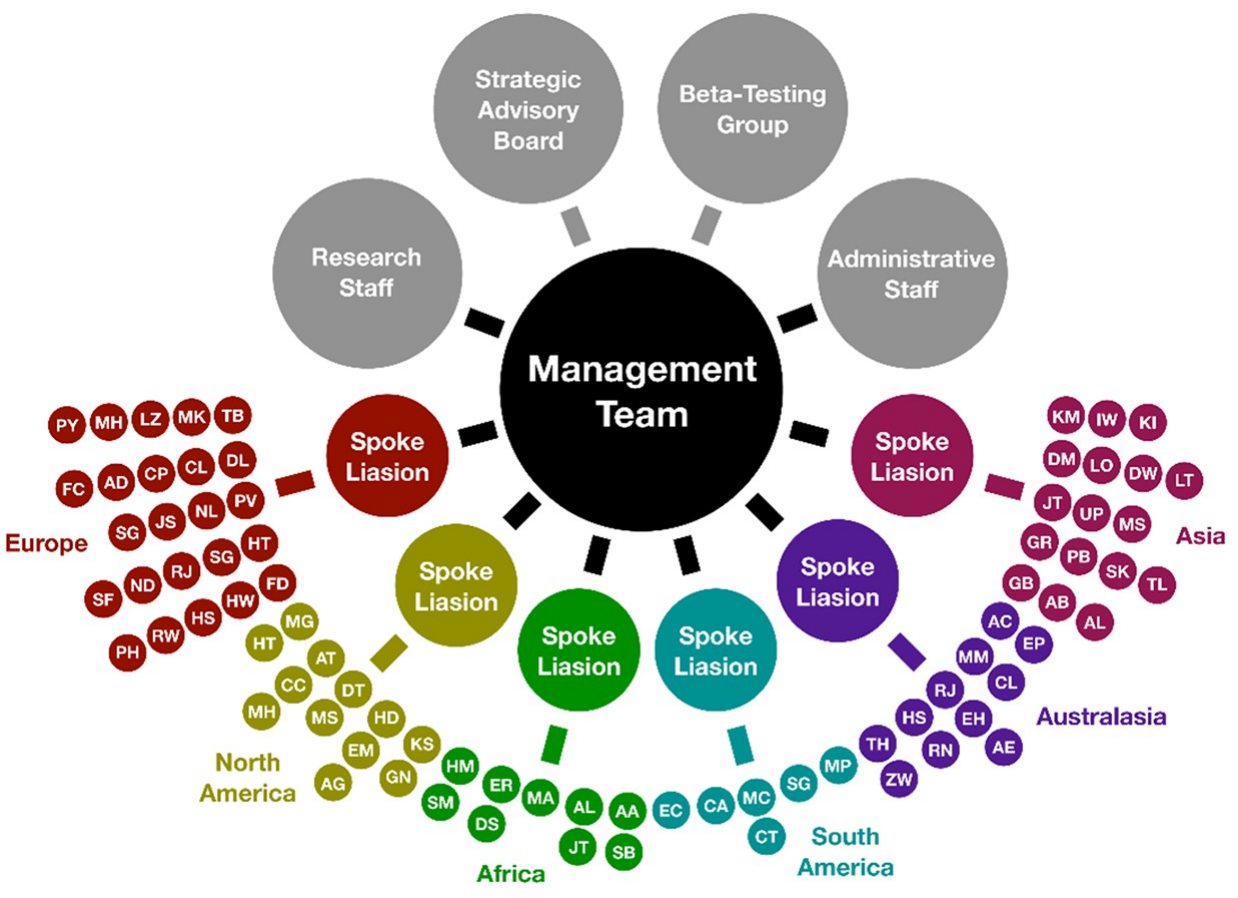
Organizational chart. Small coloured dots represent 80 academic institutions, 30 of which already participated in the June-July 2023 project, and 50 of which have since agreed to join. Each institution has a local representative or ‘Spoke Rep’; initials of Spoke Reps are included in the figure.

During August-October 2023 we expanded the network of institutions considerably, from 30 to 80 (Fig 10). This larger network contains *c*.50,000 scientists. The primary motivations are:

(i) The new network is more internationally representative: 24 institutions in Europe, 12 in North America, 6 in South America, 17 in Asia, 9 in Africa, and 12 in Australasia. This will help to ensure that inferences from the sample to the global population are reliable, and confidence interval calculations can be trusted.
(ii) The new network allows for significant survey size even when quite specific questions are asked of the data, e.g. “Is there a strong consensus amongst earth scientists in Asia?”
(iii) In a larger network, survey experiments can be conducted, where each sub-group survey within the experiment is large enough to count as a significant survey in its own right.

Regarding motivation (iii), we emphasise that a huge range of survey experiments become possible with the new network. One can make countless adjustments to the methodology, to ascertain how these adjustments affect important variables including agreement response and response rate. Of the many possible survey experiments worth considering, we mention just two here. First, it would be interesting to compare scientists’ personal views with their impression of the general consensus; in this way, we could explore the phenomenon or ‘pluralistic ignorance’ or ‘preference falsification’ [45]. Second, it would be interesting to split the population of scientists in two, running two surveys in parallel, but with one difference: inclusion or omission of a slider for the scientist to self-report their degree of expertise vis-à-vis the statement in question. If a slider can be added without harming the response rate and opt-out rate, it would provide valuable information, and might also allow us to include potentially relevant academics more freely.

Moving forward, another thing we plan is substantial consultation with experts regarding the language, culture, and politics of participating countries and regions. Our experience with CONICET shows clearly the importance of such consultation, and its potential impact on survey results. Among other things, such experts would offer an insight regarding the extent to which we should expect scientists to respond according to their personal (and perhaps private) view, as opposed to the view they are expected to have (or think they are expected to have). If we have reason to believe that scientists in a certain country or region are largely not offering their personal view, we would carefully consider whether to include data from that region in our survey.

We also comment here on the flexibility of the proposed methodology going forward. Flexibility along various dimensions is crucial, since the sphere of electronic communication is rapidly evolving. As things stand in 2024, the vast majority of scientists continue to use email on a daily basis, and email remains the primary method of communication amongst scientists. In addition, as things stand, most scientsts are comfortable with their email address being in the public domain. If one or both of these change in the near future, that needn’t destroy the proposed methodology; rather, the methodology would need to be adjusted accordingly. It will always remain the case that academics at an institution will have a convenient way of communicating with each other. Thus, a spoke rep in our network would still be able to send survey invitations to scientists at her/his institution. Precisely how this would work can only be a matter of speculation at this point in time, and our feeling is that emailing will remain feasible for many years to come.

Finally we note that, were this endeavour to properly take off on a large, international scale, as proposed, it would need to be run by an international organisation. This is primarily due to the fact that otherwise concerns could arise regarding any one country dominating the questions asked, the procedure, or having early access to the resultant data.

## Conclusion

Policymakers, NGOs, laypersons, and others, often ask the question, “Is there a scientific consensus?”, regarding a particular subject matter. Assuming the subject matter is genuinely *scientific*, this seems like an important question to ask, and answer. If scientists are united in their opinion, that is considered significant, and can be a good basis for particular actions. If scientists are divided, that signals that we are dealing with an ‘open’ question, with reasonable opinions on either side, and (probably) various divergent ‘open’ courses of action.

The study described here shows how it is possible to establish a global network to quickly ascertain scientific opinion regarding selected statements, on a large international scale, with high response rate, low opt-out rate, and in a way that incurs little survey fatigue, thus allowing for significant repeatability. Assuming periodic introduction of new spokes to the network, indefinite repeatability becomes realistic.

At the same time, a binary yes/no approach to scientific consensus has obvious problems, and the very question “Is there a scientific consensus on this matter?” warrants scrutiny. There are various different methodologies for assessing scientific opinion, and the degree of ‘consensus’, if any, will vary with method. Even within a single-statement, five-point Likert scale, survey methodology, there are different options for deriving an agreement score from the data (recall Fig 9). We have suggested that one does better to ignore the idea of an agreement score, instead setting a baseline for a particular methodology and assessing future survey results comparatively, with respect to that baseline. This is a realistic approach on our particular methodology, because of the possibility of significant, or even indefinite, repeatability: it is crucial that one doesn’t exhaust one’s population of scientists in the course of setting the baseline.

Before closing, it would be remiss not to mention optimistic scenarios. Notwithstanding backfire effects in some groups, in some contexts (e.g. [46, 47]), and doubts concerning the extent to which ‘consensus messaging’ influences people [48], it remains possible that scientific community opinion data, collected on a large international scale as envisioned here, could sway public opinion significantly, on a range of important topics (cf. [49]). This could then make viable implementation of policies (e.g. tackling climate change) that were previously too controversial for policymakers to take seriously. Consensus data will not be welcomed by all; far from it. But for the sake of those who welcome it, and wish to form opinions in light of it, relevant data should be made available.

## Supporting information

S1 Data (XLSX)

## References

[1] Cook J, Nuccitelli D, Green SA, Richardson M, Winkler B, Painting R, et al. Quantifying the consensus on anthropogenic global warming in the scientific literature. Environmental research letters. 2013;8(2):024024.

[2] Myers KF, Doran PT, Cook J, Kotcher JE, Myers TA. Consensus revisited: quantifying scientific agreement on climate change and climate expertise among Earth scientists 10 years later. Environmental Research Letters. 2021;16(10):104030.

[3] Jacobi CJ, Varga PJ, Vaidyanathan B. Aesthetic experiences and flourishing in science: A four-country study. Frontiers in Psychology. 2022;13: doi: 10.3389/fpsyg.2022.923940 36017445 PMC9396270

[4] Allum N, Reid A, Bidoglia M, Gaskell G, Aubert-Bonn N, Buljan I, et al. Researchers on research integrity: a survey of European and American researchers [version 1; peer review: 3 approved]. F1000Research. 2023;12:187. doi: 10.12688/f1000research.128733.1 37455853 PMC10349267

[5] Ackerman G, Behlendorf B, Baum S, Peterson H, Wetzel A, Halstead J. The Origin and Implications of the COVID-19 Pandemic: An Expert Survey. Global Catastrophic Risk Institute Technical Report 24–1 (February 2024).

[6] Van der Linden SL, Leiserowitz AA, Feinberg GD, Maibach EW. The Scientific Consensus on Climate Change as a Gateway Belief: Experimental Evidence. PLoS ONE. 2015;10(2):e0118489. doi: 10.1371/journal.pone.0118489 25714347 PMC4340922

[7] Bartoš V, Bauer M, Cahlíková J, Chytilová J. Communicating doctors’ consensus persistently increases COVID-19 vaccinations. Nature. 2022;606(7914):542–9. 35650433 10.1038/s41586-022-04805-y PMC9200639

[8] Duffy B, Malcolm F, May G, Hewlett K, Haggar T. Public perceptions on climate change. 2022. https://www.kcl.ac.uk/policy-institute/assets/peritia-climate-change%E2%80%8B.pdf

[9] Dalkey N, Helmer O. An Experimental Application of the DELPHI Method to the Use of Experts. Management Science. 1963;9(3): doi: 10.1287/mnsc.9.3.458

[10] Jorm AF. Using the Delphi expert consensus method in mental health research. Australian & New Zealand Journal of Psychiatry. 2015;49(10):887–97. doi: 10.1177/0004867415600891 26296368

[11] Alfano M, Vickers P. Data and Code for ‘Development of a novel methodology for ascertaining scientific opinion and extent of agreement’. OSF; 2024. 10.17605/OSF.IO/R4SY2. PMC11623554 39642116

[12] Vickers P. Identifying Future-Proof Science. Oxford: Oxford University Press; 2022.

[13] Levy N. Bad Beliefs. Oxford: Oxford University Press; 2022. 35167201

[14] Ballantyne N. Epistemic Trespassing. Mind. 2019;128(510):367–95.

[15] Narayanan A, Shmatikov V. Robust De-anonymization of Large Sparse Datasets. 2008 IEEE Symposium on Security and Privacy (sp 2008), Oakland, CA, USA. 2008:111–25.

[16] Ohm P. Broken Promises of Privacy: Responding to the Surprising Failure of Anonymization. UCLA Law Review. 2010;57:1701; U of Colorado Law Legal Studies Research Paper No. 9–12.

[17] Rocher L, Hendrickx JM, de Montjoye YA. Estimating the success of re-identifications in incomplete datasets using generative models. Nat Commun. 2019;10:3069. doi: 10.1038/s41467-019-10933-3 31337762 PMC6650473

[18] Mitchell Finnigan S, Sheppard J, Vickers P. IASCPolls: The Institute for Ascertaining Scientific Consensus Polling Platform. Zenodo: https://zenodo.org/records/8155054; 2023.

[19] McFadden BR. Examining the Gap between Science and Public Opinion about Genetically Modified Food and Global Warming. PLoS ONE. 2016;11(11):e0166140. doi: 10.1371/journal.pone.0166140 27829008 PMC5102371

[20] Kerr JR, Wilson MS. Changes in perceived scientific consensus shift beliefs about climate change and GM food safety. PLoS ONE. 2018;13(7):e0200295. doi: 10.1371/journal.pone.0200295 29979762 PMC6034897

[21] Rubinstein A. Response time and decision making: An experimental study. Judgment and Decision Making. 2013;8(5):540–51.

[22] Geng S. Decision Time, Consideration Time, and Status Quo Bias. Economic Enquiry. 2016;54(1): 433–49.

[23] Schotter A, Trevino I. Is response time predictive of choice? An experimental study of threshold strategies. Exp Econ. 2021;24:87–117.

[24] Stegenga J. Fast Science. British Journal for the Philosophy of Science. Forthcoming: https://www.journals.uchicago.edu/doi/10.1086/729617.

[25] Lynas M, Houlton BJ, Perry S. Greater than 99% consensus on human caused climate change in the peer-reviewed scientific literature. Environmental Research Letters. 2021;16:114005.

[26] Powell J. Scientists Reach 100% Consensus on Anthropogenic Global Warming. Bulletin of Science, Technology & Society. 2017;37(4):183–4.

[27] Blasius J, Thiessen V. The Use of Neutral Responses in Survey Questions: An Application of Multiple Correspondence Analysis. Journal of Official Statistics. 2001;17:351–67.

[28] Krosnick JA, Fabrigar LR. Designing Rating Scales for Effective Measurement in Surveys. In: Lyberg L, Biemer P, Collins M, De Leeuw E, Dippo C, Schwarz N, Trewin D, editors. Survey Measurement and Process Quality. New York: Wiley; 1997. pp. 141–64.

[29] Frankel HR. The Continental Drift Controversy. Cambridge: Cambridge University Press; 2012.

[30] Litman L, Rosen Z, Rosenzweig C, Weinberger-Litman SL, Moss AJ, Robinson J. Did people really drink bleach to prevent COVID-19? A tale of problematic respondents and a guide for measuring rare events in survey data. medRxiv. 2021; doi: 10.1101/2020.12.11.20246694 PMC10321604 37406017

[31] Lopez J, Hillygus DS. Why So Serious?: Survey Trolls and Misinformation (March 14, 2018). https://ssrn.com/abstract=3131087 or 10.2139/ssrn.3131087.

[32] Liu M, Keusch F. Effects of Scale Direction on Response Style of Ordinal Rating Scales. Journal of Official Statistics. 2017;33(1):137–54.

[33] Dellsén F. Consensus versus Unanimity: Which Carries More Weight? British Journal for the Philosophy of Science. Forthcoming: https://www.journals.uchicago.edu/doi/10.1086/718273.

[34] Malaspina C. An Epistemology of Noise. Bloomsbury Academic Publishing; 2018.

[35] Kahneman D, Sibony O, Sunstein CR. Noise: A Flaw in Human Judgement. HarperCollins Publishers; 2021.

[36] Brendel E, Jäger C. Contextualist Approaches to Epistemology: Problems and Prospects. Erkenntnis. 2004;61(2/3):143–72.

[37] Rysiew P. Epistemic Contextualism. In: Zalta EN, Nodelman U, editors. The Stanford Encyclopedia of Philosophy (Winter 2023 Edition); 2023.

[38] Tabossi P, Fanari R, Wolf K. Processing idiomatic expressions: Effects of semantic compositionality. Journal of Experimental Psychology: Learning, Memory, and Cognition. 2008;34(2):313–27. doi: 10.1037/0278-7393.34.2.313 18315408

[39] Weinberg JM, Nichols S, Stich S. Normativity and epistemic intuitions. Philosophical Topics. 2001;29:429–60.

[40] Machery E, Stich S, Rose D, Chatterjee A, Karasawa K, Struchiner N, et al. Gettier Across Cultures. Noûs. 2017;51:645–64.

[41] Cook J, Oreskes N, Doran P, Anderegg WRL, Verheggen B, Maibach EW, et al. Consensus on consensus: A synthesis of consensus estimates on human-caused global warming. Environmental Research Letters. 2016;11(4):048002.

[42] Silberzahn R, Uhlmann EL, Martin DP, Anselmi P, Aust F, Awtrey E, et al. Many Analysts, One Data Set: Making Transparent How Variations in Analytic Choices Affect Results. Advances in Methods and Practices in Psychological Science. 2018;1(3):337–56.

[43] Rietdijk N, Archer A. Post-Truth, False Balance and Virtuous Gatekeeping. In: Snow N, Vaccarezza MS, editors. Virtues, Democracy, and Online Media: Ethical and Epistemic Issues. Routledge; 2021.

[44] Hilbeck A, Binimelis R, Defarge N, Steinbrecher R, Székács A, Wickson F, et al. No scientific consensus on GMO safety. Environmental Sciences Europe. 2015;27(4): doi: 10.1186/s12302-014-0034-1

[45] Kuran T. Private Truths, Public Lies: The Social Consequences of Preference Falsification. Harvard: Harvard University Press; 1997.

[46] Ma Y, Dixon G, Hmielowski JD. Psychological reactance from reading basic facts on climate change: The role of prior views and political identification. Environmental Communication. 2019;13(1):71–86.

[47] Dixon G, Hmielowski J, Ma Y. More Evidence of Psychological Reactance to Consensus Messaging: A Response to van der Linden, Maibach, and Leiserowitz (2019). Environmental Communication. 2019;17(1):9–15.

[48] Landrum A, Slater M. Open Questions in Scientific Consensus Messaging Research. Environmental Communication. 2020;14(8):1033–46.

[49] Lewandowsky S, Gignac G, Vaughan S. The pivotal role of perceived scientific consensus in acceptance of science. Nature Climate Change. 2013;3:399–404.

